# Differential Responses of the Coral Host and Their Algal Symbiont to Thermal Stress

**DOI:** 10.1371/journal.pone.0026687

**Published:** 2011-10-24

**Authors:** William Leggat, Francois Seneca, Kenneth Wasmund, Lubna Ukani, David Yellowlees, Tracy D. Ainsworth

**Affiliations:** 1 School of Pharmacy and Molecular Sciences, James Cook University, Townsville, Australia; 2 Australian Research Council Centre of Excellence for Coral Reef Studies, James Cook University, Townsville, Australia; King Abdullah University of Science and Technology, Saudi Arabia

## Abstract

The success of any symbiosis under stress conditions is dependent upon the responses of both partners to that stress. The coral symbiosis is particularly susceptible to small increases of temperature above the long term summer maxima, which leads to the phenomenon known as coral bleaching, where the intracellular dinoflagellate symbionts are expelled. Here we for the first time used quantitative PCR to simultaneously examine the gene expression response of orthologs of the coral *Acropora aspera* and their dinoflagellate symbiont *Symbiodinium*. During an experimental bleaching event significant up-regulation of genes involved in stress response (HSP90 and HSP70) and carbon metabolism (glyceraldehyde-3-phosphate dehydrogenase, α-ketoglutarate dehydrogenase, glycogen synthase and glycogen phosphorylase) from the coral host were observed. In contrast in the symbiont, HSP90 expression decreased, while HSP70 levels were increased on only one day, and only the α-ketoglutarate dehydrogenase expression levels were found to increase. In addition the changes seen in expression patterns of the coral host were much larger, up to 10.5 fold, compared to the symbiont response, which in all cases was less than 2-fold. This targeted study of the expression of key metabolic and stress genes demonstrates that the response of the coral and their symbiont vary significantly, also a response in the host transcriptome was observed prior to what has previously been thought to be the temperatures at which thermal stress events occur.

## Introduction

Successful symbiotic interactions depend upon the integration of gene expression and metabolism of both the host and symbiont, including successful responses to environmental stress. Currently a variety of anthropogenic threats, including increased temperature, ocean acidification, and eutrophication threaten the integrity of the coral holobiont, which consists of the coral host, intracellular dinoflagellate (*Symbiodinium*) and associated prokaryotes and viruses [1]. Coral bleaching is one of the most dramatic examples of the stress induced dysfunction of a symbiosis, where the *Symbiodinium* are expelled from their coral host, or lose their photosynthetic pigments, in response to a variety of stressors including high [2] and low [3] temperatures, acidification [4] and disease [5].

The initial point of thermal damage in the coral holobiont has not been determined, however, a variety of *Symbiodinium* related causes have been proposed including dysfunction of the D1 protein of photosystem II [6], the repair machinery of D1 [7], Rubisco [8], the carbon concentrating mechanism [8] and the light harvesting complexes [9]. No matter the primary cause, the thermal damage to *Symbiodinium* leads to the production of reactive oxygen species which, it has been proposed, damages both the symbiont and the coral host (for review see [10]) and leads to the breakdown of the symbiosis through one of a variety of cellular mechanisms [11]. Recently, there has been a much greater emphasis on the response of the coral host to thermal stress and how it can mitigate the impact of elevated temperatures (for reviews see [12]). Apart from visual differences in pigmentation, one of the most widely used methods for detecting the early signs of bleaching is through the use of a Pulse Amplitude Modulated Flurometer (PAM) which measures the photosynthetic capacity of photosystem II (PSII) [13]. However it has recently been demonstrated that the host cellular response, in this case the induction of apoptosis, can be detected before visual signs of bleaching or significant changes in PSII photochemistry are noted [14], [15].

Recent advances in our knowledge of both the coral [16], [17], [18] and *Symbiodinium* transcriptome [19], [20], [21] are now enabling us to probe the transcriptomic responses of the coral host [22], [23] and algal symbiont [24], [25] to a variety of environmental challenges. However, as yet no study has simultaneously examined the responses of both the coral host and *Symbiodinium*, with a key part of this symbiosis, the metabolic reliance of each partner on the other, being neglected. In particular there are no studies on the regulation of some of the basic metabolic pathways, including glycolysis, tricarboxylic acid cycle (TCA) and fatty acid metabolism and how they may be affected in response to increased temperatures. Given that the coral host can obtain up to 90% of its energy requirements from *Symbiodinium* [26], any alterations in the metabolic function of either, or both, partners has the potential to significantly alter the energy budget of the holobiont.

The coral *Acropora aspera* is found on reef flats and shallow lagoons, at Heron Island there are four distinct morphologies with different colours (blue, green, cream and light blue) which have signficantly different physiologies [27]. Water temperatures can fluctuate significantly between day and nighttime with values regularly more than 4°C per day at 0.3 m depth (IMOS, 2011, Heron Island, http://data.aims.gov.au/aimsrtds/datatool.xhtml?channels=&checked=&site=130). Interestingly, the *A. aspera* morph, which expresses the greatest amount of host derived green fluorescent proteins (blue morph), is the the most thermally susceptible when compared to the cream and light blue morph, with large loss of symbionts and larger decreases in dark adapted yields of PSII at the same temperatures [27]. Significant decreases in the dark adpated yield were only seen in the light blue and cream morph when temperatures exceeded 32°C for extended periods. In another study decreases in *Symbiodinium* numbers and PSII yields were only seen when tan *A. aspera* had been exposed to 34°C for 5 days and 3 days respectively [28].

This study focussed on the response of a number of gene orthologs encoding metabolic and stress proteins in both the coral host and the resident *Symbiodinium* population during a thermal stress that simulated the lead up to a bleaching event in the cream morph of *A. aspera*. We find that the response of *Symbiodinium* differs markedly to that of the coral host, exhibiting few transcriptional changes as the temperature increased.

## Results

**Figure 1:**
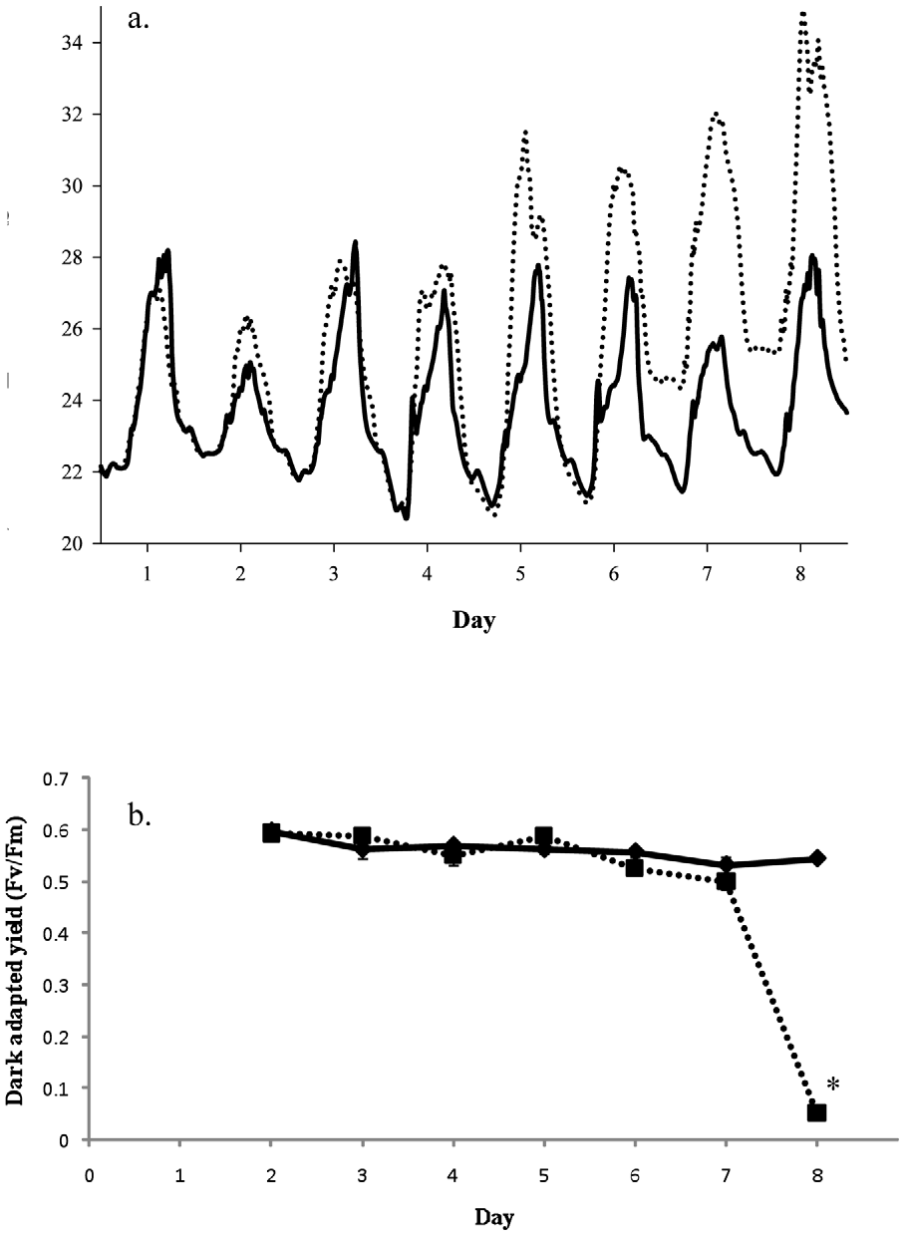
Temperature profile and dark-adapted yield for *A. aspera* during the course of the experiment. (a) Temperature represents the average of the three experimental tanks (…) and three control tanks (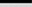). (b) Dark adapted yield of heated (▪) and control (♦) corals was measured at 18:30 each day after the corals had been dark-adapted for 30 minutes, n = 9, error bars represent standard errors, * represents significant differences between controls and treatments (*p*<0.05).

Temperatures in the heated tanks were increased from a midday temperature of approximately 27°C to a midday value of 34°C over the 8 days of the experiments (Figure 1a). Analysis of the dark-adapted yield found that there were significant differences between treatments (*p*<0.001, df = 1), day (*p*<0.001, df = 6) and temperature × day (*p*<0.001, df = 6) (Figure 1b). A subsequent Bonferonii post-hoc analysis found that these differences were driven exclusively by a significant difference between controls and treatment on day 8 (*p*<0.001). Paling of the corals was only observed on the final day.

### Heat shock protein response

**Figure 2:**
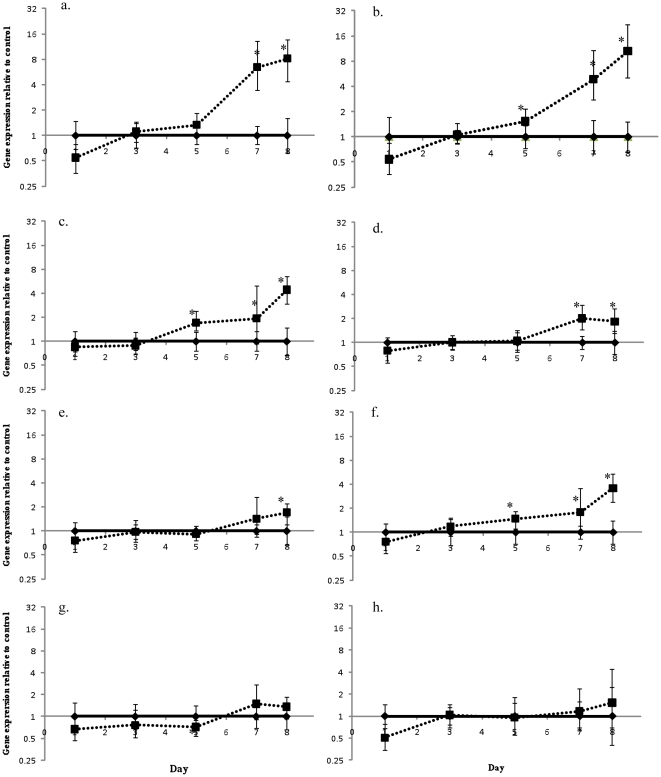
Changes in *A. aspera* gene expression during a simulated bleaching event. Changes in gene expression of heated *A. aspera* nubbins compared to control nubbins on the same day for (a) HSP70, (b) HSP90, (c) GAPDH, (d) α-ketoglutarate dehydrogenase, (e) glycogen synthase, (f) glycogen phosphorylase, (g) glutamine synthetase and (h) malonyl Co-A acyl transferase. Controls (♦) have relative expression values of while heated tanks (▪) are changes in gene expression relative to controls on that day. Error bars represent standard error, n = 6 for each treatment, * represents significant differences between controls and treatments (*p*<0.05).

**Figure 3:**
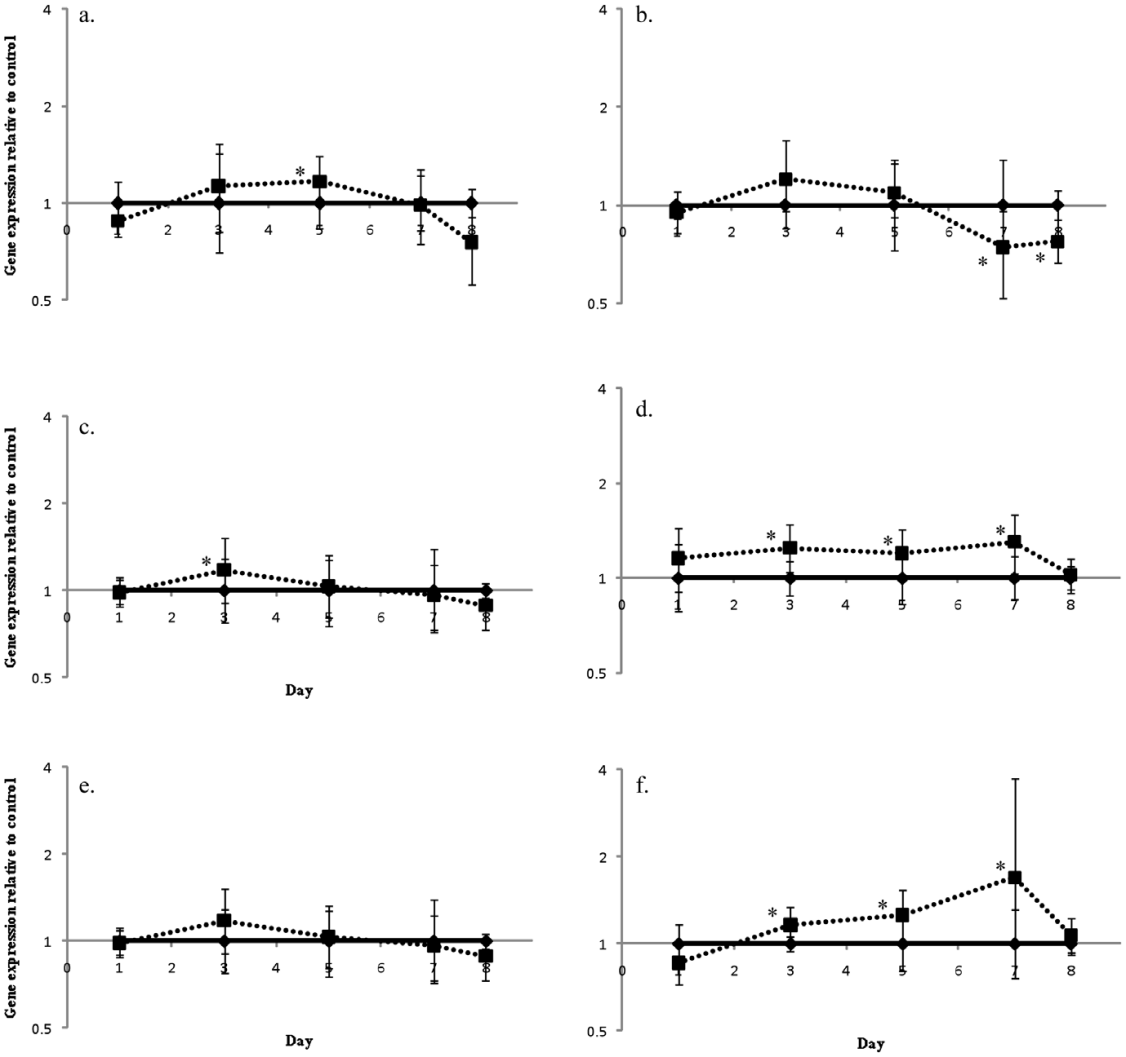
Changes in *Symbiodinium* gene expression during a simulated bleaching event. Changes in gene expression in *Symbiodinium* of heated *A. aspera* nubbins compared to control nubbins on the same day for (a) HSP70, (b) HSP90, (c) GAPDH, (d) α-ketoglutarate dehydrogenase, (e) glutamine synthetase and (f) malonyl Co-A acyl transferase. Controls (♦) have relative expression values of while heated tanks (▪) are changes in gene expression relative to controls on that day. Error bars represent standard error, n = 6 for each treatment, * represents significant differences between controls and treatments (*p*<0.05).

The response of both HSP70 and HSP90 genes was determined for the coral and *Symbiodinium* over the course of the experiment (Figure 2a,b and 3a,b). In the coral host the expression of both HSP70 and HSP90 increased when exposed to increased temperature and were significantly elevated from day 7 and 5 respectively. On day 5, when the heated treatment was 4°C above controls HSP70 levels increased, although not statistically significantly, 1.3 fold over controls (*p* = 0.119) on day 5, 6.4 fold on day 7 (*p* = 0.001) and 8.2 fold (*p* = 0.001) on day 8. Significant and progressive increases in HSP90 levels were detected on day 5 (1.5-fold, *p* = 0.022), day 7 (4.9-fold, *p*<0.0001) and day 8 (10.5-fold, *p* = 0.001).

In contrast, *Symbiodinium* HSP70 only increased significantly on day 5 (1.2-fold, *p* = 0.039) and HSP90 levels were found to significantly decrease, on day 7 where a 0.74-fold decrease (*p* = 0.036) was observed and on day 8 with a 0.77-fold decrease (*p* = 0.008) (Figure 3a,b).

### Carbon metabolism

In addition to examining heat shock protein responses, a series of orthologs of genes involved in glycolysis, the TCA cycle and fatty acid synthesis were examined in the coral host and *Symbiodinium*. Generally, expression of those genes involved in carbon metabolism was up-regulated in the coral as temperatures increased. GAPDH expression increased on day 5 (1.7-fold, *p* = >0.0001), 1.9-fold on day 7 (*p* = 0.048) and 4.4-fold on day 8 (*p* = 0.001) and α-ketoglutarate dehydrogenase expression significantly increased on day 7 (2.0-fold, *p* = 0.001) and day 8 (1.8-fold, *p* = 0.009) (Figure 2c,d)). *Symbiodinium* GAPDH was slightly elevated but only on day 3 (by 1.3-fold, *p*>0.001) while α-ketoglutarate was up-regulated on days 3 (1.2-fold, *p* = 0.02), 5 (2.0-fold, *p* = 0.048) and 7 (1.3-fold, *p* = 0.027) (Figure 3c,d). Two additional genes coding fro enzymes involved in carbon metabolism, glycogen synthase which is a key enzyme involved in glycogen synthesis, and glycogen phosophorylase which is involved in glycogen catabolism, could only be examined in the coral (The equivalent algal genes, starch synthase and starch phosphorylase, were not found in the available *Symbiodinium* transcriptome data.) Surprisingly, given that these genes encode enzymes that are involved in opposing metabolic pathways, the expression of both increased with increasing temperature; in the case of the glycogen synthase gene expression increased on day 8 (1.7-fold, *p* = 0.007) while for glycogen phosphorylase gene expression increased earlier, on days 5, 7 and 8, and to a greater extent (1.5-fold, *p* = 0.005, 1.8-fold, *p* = 0.010 and 3.6-fold, *p*<0.001) (Figure 2e,f).

### Nitrogen metabolism and fatty acid synthesis

One of the fundamental reasons for the success of the coral symbiosis is the tight cycling of nitrogen. Expression of the gene encoding glutamine synthetase (GS) was followed in both *A. aspera* and *Symbiodinium*. In contrast to the genes involved in coral carbon metabolism no consistent changes in gene expression were observed. Only on day 5 was there a significant difference seen in GS between the treatments and controls (0.72-fold decrease, *p* = 0.034) (Figure 2g). Throughout the course of the experiment *Symbiodinium* GS expression was unaffected by the increasing temperature (Figure 3e).

The expression of malonyl Co-A acyl transferase was followed as a proxy for fatty acid synthesis. As with GS, host expression of malonyl Co-A acyl transferase was unaffected by temperature (Figure 2h). In contrast, expression in *Symbiodinium* was significantly elevated above controls from day 3 until day 7 (day 3, 1.3-fold *p* = 0.031, day 5, 1.3-fold *p* = 0.025, day 7, 1.7-fold *p* = 0.04) (Figure 3f).

### Daily changes in gene expression

**Figure 4:**
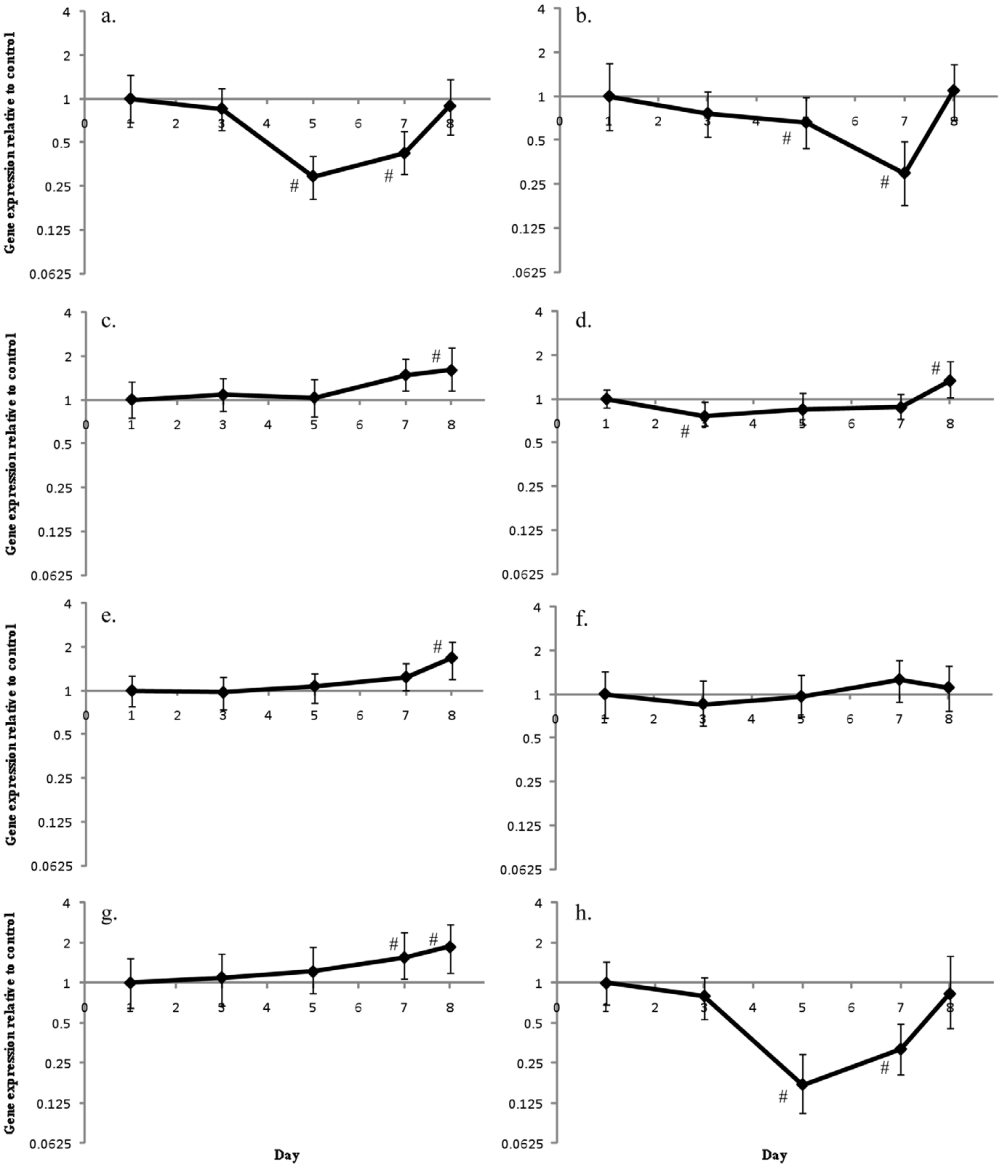
Natural variations in *A. aspera* gene expression. Changes in gene expression of control *A. aspera* nubbins compared to expression of control nubbins on day one for (a) HSP70, (b) HSP90, (c) GAPDH, (d) α-ketoglutarate dehydrogenase, (e) glycogen synthase, (f) glycogen phosphorylase, (g) glutamine synthetase and (h) malonyl Co-A acyl transferase. Error bars represent standard error, n = 6 for each treatment, # represents significant differences from day 1 expression (*p*<0.05).

Over the course of the experiment significant natural differences in gene expression were detected in control corals, when compared to control controls on day 1, however there was no consistent pattern of expression changes (Figures 4a–h). α-ketoglutarate dehydrogenase was found to be significantly different from controls on day 1, day 3 (0.76-fold, *p* = 0.023) and day 8 (1.9-fold, *p* = 0.01), while HSP70 (day 5, 0.29-fold, *p*<0.0001; day 7, 0.42-fold, *p*<0.001), HSP90 (day 5, 0.66-fold, *p* = 0.047; day 7, 0.30-fold, *p* = 0.003) and malonyl Co-A acyl transferase (day 5, 0.17-fold, *p* = 0.002; day 7, 0.32-fold, *p* = 0.002) were all significantly down-regulated on days 5 and 7 when compared to day 1 controls. Finally, GS (day 7, 1.6-fold, *p* = 0.018; day 8 1.9-fold, *p* = 0.01) was up-regulated on days 7 and 8, while glycogen synthase (1.7-fold, *p* = 0.006) and GAPDH (1.6-fold, *p* = 0.014) were up-regulated on day 8.

**Figure 5:**
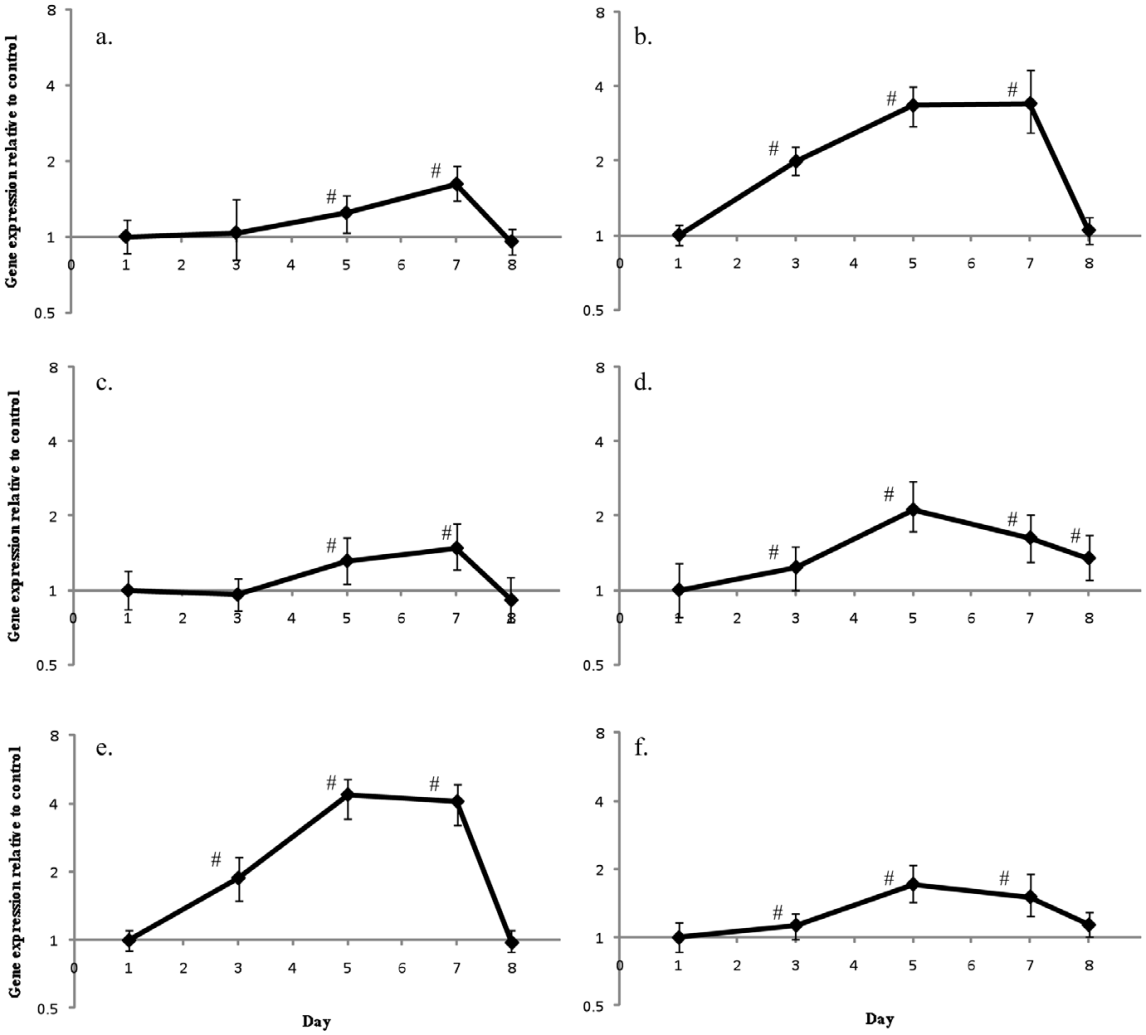
Natural changes in *Symbiodinium* gene expression. Changes in gene expression of *Symbiodinium* from control *A. aspera* nubbins compared to expression of control nubbins on day one for (a) HSP70, (b) HSP90, (c) GAPDH, (d) α-ketoglutarate dehydrogenase, (e) glutamine synthetase and (f) malonyl Co-A acyl transferase. Error bars represent standard error, n = 6 for each treatment, # represents significant differences from day 1 expression (*p*<0.05).

In contrast to the patterns seen in the coral host, there was a consistent pattern of up-regulation of gene expression in *Symbiodinium* when compared to day 1 controls (Figure 5a–f). On day 3 HSP90 (2.0-fold, *p* = 0.002), GS (1.9-fold, *p* = 0.002), malonyl Co-A acyl transferase (1.1-fold, *p* = 0.048) and α-ketoglutarate dehydrogenase (1.2-fold, *p* = 0.033) were all up-regulated. Expression of all the *Symbiodinium* genes examined in this study was found to significantly increase expression on days 5 and day 7. Subsequently, expression of all the genes, except malonyl Co-A acyl transferase (1.1-fold, *p* = 0.041) and α-ketoglutarate dehydrogenase (1.3-fold, *p*<0.0001) decreased on day 8.

## Discussion

This study represents the first time that the transcriptomic response of the coral host and their symbionts have been examined simultaneously. Recently we reported it has been found that there are a series of responses, including decreases in tissue thickness and host cell apoptosis, that occur in the symbiosis prior to the loss of symbionts or a significant lasting decline in dark adapted yield [14]. This report extends these studies by examining transcriptomic changes in both symbiotic partners during this pre-bleaching period. It is well established that increases in seawater temperatures of only a degree above long-term summer maxima lead to coral bleaching. Over the course of this experiment the temperature was increased from approximately 27°C to 34°C over a period of 8 days (Figure 1a), simulating a bleaching event. A significant decline in F_v_/F_m_ was not observed until day 8 when seawater temperatures had been at 34°C for 2 days. Interestingly however, significant changes in the expression of metabolic genes were seen in the coral host prior to this. It is also noteworthy that, from the expression patterns, the transcriptomic levels response of the coral host and *Symbiodinium* are significantly different, with very little change in the expression of metabolic genes in *Symbiodinium* even when significant declines in photosynthetic parameters are observed.

### Host gene expression patterns

A variety of studies have previously demonstrated an increase in HSP90 and/or HSP70 protein levels when corals are exposed to increased thermal stress (e.g., [29], [30], [31], [32]). Increases in transcript abundance in the host have also been detected under thermal stress using microarrays for HSP90 in *Montastraea faveolata* [33] and quantitative PCR for HSP70 in *Acropora millepora* [23]. In both these cases transcript abundance was measured in corals that were classified as moderately bleached, unlike experimental conditions used in this study. In *M. faveolata*, HSP90 expression was increased 1.3-fold, in contrast to the 10 fold increase observed in this study. In *A. millepora* the average increase in HSP70 transcript levels was 12±20-fold, which is slightly higher than that observed in this study (8.2-fold). The pattern of increase in HSP transcript abundance seen in our results clearly indicates that the coral host gene expression responds to sub-bleaching temperature. While it has not been documented before in the coral host, it is not surprising that small changes in temperature modify HSP expression levels, as this has been well characterised in other systems. This confirms that previous studies conducted at or above bleaching temperatures miss the early host responses that may have a significant influence on bleaching outcomes.

Surprisingly in microarray studies that have examined the bleaching response of the coral host, only genes encoding proteins involved in the electron transport chain have been identified as changing. For example NADH-ubiquinone oxidoreductase, a protein involved in complex I of the electron transport chain, was found to be down-regulated early during thermal stress [33]. Here, in contrast, genes encoding proteins involved in carbon metabolism (GAPDH, α-ketoglutarate dehydrogenase, glycogen synthase, glycogen phosphorylase) increased significantly over the course of the experiment. The up-regulation of those genes coding for enzymes involved in carbon metabolism suggests that there is an increased requirement for carbon substrates for respiration. This is consistent with the increase in whole coral respiration, and corresponding decrease in photosynthesis:respiraton rates reported by Coles and Jokiel [34], [35] when temperature is increased. In *Porties lutea* an increase in temperature from 26.8°C to 29.7°C resulted in a 35% increase in respiration [35]. The expression of genes, and presumably proteins, involved in carbon metabolism may account for the increased respiration rates.

The expression of GS, the key enzyme linking the carbon and nitrogen cycles, did not change with increasing temperature. While there is still some controversy as to the primary site (host or symbiont) of ammonium assimilation (for review see [36]) it has recently been suggested that the coral host utilises photosynthetically fixed carbon exported from the symbiont as the carbon skeleton for ammonium assimilation [37]. The lack of change in host GS levels seems to indicate, that although there may be changes occurring in carbon metabolism, there is no increased requirement for nitrogen assimilation, which could potentially be utilised for increased protein synthesis and growth.

Finally there was also no change in the expression of the coral host malonyl Co-A acyl transferase, which encodes an enzyme involved in fatty acid synthesis. Previous studies have found the lipid bodies in the coral host are positively correlated with *Symbiodinium* numbers [38], [39], [40]. Given that bleaching had not occurred in this experiment it is perhaps not surprising that malonyl-CoA acyl transferase expression was unchanged.

### Symbiodinium gene expression patterns

Despite there being significant changes in the expression of host genes detected by day 5 of the experiment, there were comparably few consistent changes found in *Symbiodinium* gene expression, and those changes that were observed were much smaller (in all cases less than 2 fold) than those in the coral host. We have previously demonstrated that accurate measurements of *Symbiodinium* gene expression can be made when the mRNA is less than 3% of the total holobiont RNA [24]. HSP70 levels were only slightly elevated (1.2-fold increase) on day 5 of the experiment while HSP90 levels were found to decrease to between 0.743 and 0.773 fold when compared to controls (Figure 3a). Similar changes have previously been observed in *Symbiodinium* isolated from *A. millepora* when subjected to “fast” thermal stress (8°C increase over 18 hours) and “gradual” thermal stress (7°C over 5 days). In the later 5 day experiment HSP70 levels initially increased by 39% at 3°C above ambient and then dropped by between 41% and 31% at the end of both experiments. Similarly HSP90 levels were found to decrease by 43% and 57% at the end of the experiments [41].

The largest changes in *Symbiodinium* gene expression occurred for α-ketoglutarate dehydrogenase and malonyl-CoA acyl transferase, both of which increased from day 3 to 7 of the experiment, before they returned to control levels. Unlike the coral host, there was no concomitant up-regulation of GAPDH or other *Symbiodinium* genes examined. Increases in TCA cycle enzymes, but not glycolytic enzymes, are characteristic of the use of fatty acids for respiration. Significant changes in the lipid content of *Symbiodinium* are known to occur as the corals move from a pre- to post-bleaching state, with an increase in neutral lipids after thermal stress [38]. There are two possible explanations for the changes seen in *Symbiodinium* malonyl Co-A acyl transferase. Firstly, it is possible that increased thermal stress is leading to alterations in the *Symbiodinium* membrane composition. Diaz-Almeyda et al. [42] have recently shown that the lipid composition alters in *Symbiodinium* when acclimated to elevated temperatures (24°C to 31°C) for two weeks. In *Symbiodinium* C1, a closely related strain to the C3 in this experiment, this acclimation resulted in an increase in lipid saturation and an increase in the relative proportion of C22 fatty acids extracted from the whole cell, although no other large changes were observed. A second hypothesis for the increase in malonyl Co-A acyl transferase is that increased lipids are synthesised for export to the coral host. Lipids are reported to be one of the major components by which photosynthetically fixed carbon is transported from *Symbiodinium* to the coral [43], and alterations in bleaching state have been shown to change the carbon exchange in *Symbiodinium* symbioses [44]. Examination of both host and symbiont during the lead up to bleaching should resolve this question.

The relatively small changes in *Symbiodinium* transcript levels are consistent with other studies that have examined both *Symbiodinium* [24], [41] (for review see [21]) and dinoflagellate gene expression [45], [46], [47], [48]. These studies show that there are few large gene expression changes in dinoflagellates, even under significantly different conditions. Instead it appears that much of the regulation occurs post-translationally [49], [50], [51]. This lack of change in gene expression levels in certain biochemical pathways means that it will be necessary to pursue other methodologies, such as proteomics or metabolomics [52], to gain an insight to the *Symbiodinium* response.

It is interesting to compare the small changes seen in the symbiont transcriptome observed here with another intracellular animal symbiosis, that between aphids and the bacterial symbiont *Buchnera aphidicola*. In this symbiosis the symbiont supplies the aphid host with nine essential amino acids that are not found in the aphid's sap diet. Under naturally increased temperatures the symbiont population decreases and under extreme temperature stress the *B. aphidicola* cells dies [53], [54]. Similar to *Symbiodinium*, under near lethal heat stress only a small number of genes in *B. aphidicola* demonstrated differential expression [55], and of the 20 genes that are part of the heat stress regulon [56], only 5 showed up-regulation [55]. Of the five heat shock genes which were differentially regulated, expression changes were significantly smaller (less than 10 fold) compared to other free-living bacteria subjected to similar stress (greater than 100 fold up-regulation) [55]. In those systems where gene expression does not change, any metabolic changes are likely to be a result of changes in substrate availability and/or post-translational or allosteric regulation of the enzymes themselves.

A major factor that must be taken into account in all studies of coral gene expression is the underlying variation seen between days and times, driven by either light/dark entrained gene expression or in response to the light driven symbiont photosynthesis. A recent microarray study of diel cycling in the coral host found that genes encoding the glycolytic enzymes glyceraldehyde-3-phosphate dehydrogenase, phosophoglycerate kinase and aldolase, and the electron transport chain enzyme NADH-ubiquinone oxidoreductase, were co-regulated over a 24 hour period with peak expression levels being found at midnight [57]; it was proposed that these genes were regulated in response to symbiont physiology [57]. While there were significant differences in control corals in this study following day 1 (Figure 4), these changes did not display any consistent pattern or correlation to either temperature or light levels. These would be expected to be the main drivers in a photosynthetic holobiont (Figure 4a–g). In contrast, in all *Symbiodinium* genes examined over the course of the experiment there was an increased their expression (compared to day 1 controls) before reducing by day 8 (Figure 5a–f). As with the coral, these changes were not correlated to light levels that were measured during the experiment, so the underlying factor driving these changes is still to be identified.

The genes examined in this study were chosen as they encode key metabolic proteins. In many organisms these proteins are not solely regulated by transcription but are also subject to post-translational and allosteric modifications. These mechanisms are not captured by this study and their importance cannot be assessed without isolation and characterisation of the proteins themselves.

This study highlights, that while the emphasis is now on understanding the response of the coral holobiont, the stress response of individual partners in the symbiosis may vary significantly. It is also clear from this study that measurable changes in the expression of genes in the host occurs well before the temperature required for observable bleaching are reached. Given that prior acclimation to elevated, but not bleaching temperatures, significantly modify bleaching outcomes [28], it is important to link these transcriptional changes to physiological responses and eventual bleaching outcomes. The use of other physiological measures, such as oxygen evolution, dark respiration and ^14^C labelling studies would help elucidated the response of both symbiont and host and link the transcriptome responses to physiology. A greater understanding of the molecular and cellular responses of the coral holobiont to thermal, and other, stressors will provide an additional layer of information to include in current bleaching models that might contribute to an increased accuracy in bleaching predictions. The ability to examine gene expression of the coral host and algal symbiont, and soon the prokaryote community using a metagenomic approach, is now enabling us to finally tease apart the role of each partner in this symbiosis.

## Materials and Methods

Individual coral nubbins (n = 100) were collected from three *Acropora aspera* patches (cream morph, sensu [27]) colonies situated on the reef flat of Heron Island (23°33′S, 151°54′E) in November 2008. The algal symbiont was *Symbiodinium* clade C3 (sensu LaJuenesse [58]). Corals were collected under Great Barrier Marine Park Authority permit G08/26873.1. Nubbins were randomly placed inside racks holding them in a vertical position before placing into one of six flow through 65 l aquaria at the Heron Island Research Station. Water was taken from the reef flat and filtered through a sand filter. Regrowth of tissue on the lesion could be seen after 5 days. After five days acclimation the three experimental tanks were heated by approximately 1°C per day for 6 days before they were held at the specified temperature for an additional 2 days. Over the course of the experiment temperatures in the tanks were recorded every 5 minutes using a Hobo dataloggers (Onset, Massachusetts, USA) (Figure 1a). Corals were exposed to ambient light levels over the course of the experiment. Due to a logger malfunction light levels were only recorded over the final 5 days of the experiment and during this time maximum daily light levels varied from 590 to 2445 µmol m^−2^ s^−2^. The dark-adapted yield of 10 nubbins from each tank was determined on each day of the experiment. This was done by collecting the nubbins at 18:00, just prior to sunset, dark-adapting them for 30 min and then measuring the dark-adapted yield with an Imaging Pulse Amplitude Modulating Fluorometer (IPAM; Walz, Germany).

### Sample collection and gene expression analysis

At 1800 each day of the experiment 2 nubbins from each tank were collected and snap frozen in liquid nitrogen and stored at −80°C until analysis. For RNA isolation the entire branch was removed from the freezer and then placed in a mortar that had been pre-cooled with liquid nitrogen. The entire branch was then crushed with a hydraulic press, and the resulting powder was then further ground in a mortar and pestle. mRNA was purified using a modified method of Csaszar et al [23]. After grinding 400 mg of the powder was added to 400 µl Dynabeads lysis buffer (Invitrogen, Australia), this was then vortexed for 2 min and centrifuged at 12000 g for 1 min to remove the calcium carbonate. The supernatant was then added to 200 µl of Dynabeads, which had been pre-washed in lysis buffer, and vortexed for 5 min. The beads and sample were then placed on a magnetic column for 2 minutes, after which the supernatant was discarded. The remaining beads were twice washed with 1 ml of buffer A and then washed twice in 500 µl of buffer B. Finally the mRNA was eluted from the beads by adding 25 µl of cold 10 mM Tris-HCl (pH 7.4), heating to 80°C for 2 min, placing on ice and then on the magnet. The supernatant containing the eluted mRNA was then removed. After elution the mRNA concentration was determined using a Nanodrop (Thermo Scientific, Willmington) and the quality checked using a Bioanalyzer (Agilent, California) and DNase treated with 1 unit of DNase per 100 ng mRNA as per manufacturer's protocol (Promega, USA).

cDNA synthesis was performed using the Superscript III Platinium Taq 2 step RT-PCR kit (Invitrogen, Australia), 100 ng of mRNA was added to 10 µl of 2× RT reaction mix and 2 µl Superscript III, the volume was then made up to 20 µl. cDNA was synthesised by incubating at 25°C for 10 min, 50 min at 42°C and the 5 min at 85°C, the reaction was then placed on ice, 1 µl (2 units) of RNase H was added and then incubated at 37°C for 20 min. The resulting cDNA was then diluted 1 in 20 and used for the qPCR reactions.

Primers for qPCR were designed for both the coral host and *Symbiodinium* genes that code for enzymes in key metabolic pathways (Table 1). Coral and *Symbiodinium* primers were designed for genes involved in general stress response (heat shock protein 90 (HSP90) and heat shock protein 70 (HSP70)), glycolysis (cytosolic glyceraldehyde-3-phosphate dehydrogenase (GAPDH)), the tricarboxylic acid cycle (α-ketoglutarate dehydrogenase (α-kg)), nitrogen assimilation (glutamine synthetase (GS)) and fatty acid synthesis (malonyl Co-A acyl transferase (mal-CoA)). Two additional coral genes involved in glycogen synthesis (glycogen synthase (g-syn)) and catabolism (glycogen phosphorylase (g-phos)) were also chosen; *Symbiodinium* orthologs were not available for these genes. In addition to the genes of interest adoHcyase (Ado) and ribosomal protein S7 (rpS7) were used as coral housekeeping genes [59] and β-actin and proliferating cell nuclear antigen (PCNA) for *Symbiodinium* [24]. Quantitative PCR was performed using SYBR-Green (Invitrogen), triplicate reactions were conducted in a total volume of 15 µl and consisted of 7.5 µl 2× Platinum SYBR Green qPCR Supermix, two primers for each gene (final concentration 850 nM) and 4 µl cDNA (diluted 1 in 20). Reactions were performed on a Corbett Rotorgene RG6000 using the following protocol; denaturation 10 min at 95°C, 35 cycles of 95°C for 15 s then 65°C for 30 s. At the end of the cycling a melt curve was performed from 60°C to 95°C to ensure only one peak was observed indicating no non-specific products. The efficiency of each primer set was determined using a serial dilution (between 1 in 10 and 1 in 120) of a mixed sample, in all cases the efficiency was between 0.94 and 1.10 (Table 1). To ensure specificity of the primers PCR reactions were performed using *Symbiodinium* primers with coral larval cDNA, which is *Symbiodinium* free, and coral primers with cultured cDNA sourced from cultured *Symbiodinium* (clade C1), in all cases no non-specific products were observed.

**Table 1:** Primer sequences used for quantitative PCR for both *A. aspera* and *Symbiodinium* clade C1.

Organism	Gene name	Forward Primer	Reverse Primer	Reaction efficiency	Accession number or citation
Coral	Glutamine synthetase	TTTGGATCGATGGAACAGGAGAG	TCGGCACTAAGAGGTTCGGATTC	1.10	DY579366.1
	HSP90	TTTCTTGTTGCTGACCGTGTAATAGTA	CCCCTGGGATCCTCTGTGA	0.99	DY584045.1
	HSP70	GTCGCTCTCAATCCATCAAATACT	GTCTCCCACCTTCGCTTACG	0.98	GO000475.1
	Malonyl Co-A carboxylase	GTTGGGCGGGGACACATCATC	GCAGACGTCATCAGTTCAGTAT	1.02	EZ001045.1
	α-ketoglutarate dehydrogenase	AGTTAGGCTTCATGTCCGCTTTCA	GACAATCTCACTGCCTTCAATAACTGC	1.00	EZ012855.1
	Glycogen phosphorylase	CTCAGGAAAGCCATCAATCAAATCAGG	TGCATCAAAGTCGGCCATAAGAAGG	1.01	EZ031953.1
	Glycogen synthase	TCAAATCTGCCGAGGAATCAATCAA	AGTCGCTCCGTTCTGTTTCTCTGG	1.01	GW213759.1
	GAPDH	GAGGCTGGTGCAGATTTTGT	TGACTTTCTTGGCTCCACCT	1.04	EZ026309.1
	rpS7	AGCAAAGGAGGTTGATGTGG	GACGGGTCTGGATCTTTTGA	0.97	[59]
	AdoHcyase	AAGAAGACAAACATCAAGCCTCA	CACATCCAAGGTTCACAAGACG	0.94	[59]
*Symbiodinium*	Glutamine synthetase	GAGGAAACCGAGGCCCGTCAGGAAGTGA	AGCAGGCAAGAAGCCGGTCTCGGTCATC	1.00	JF915366
	HSP90	GCTTGAGTTCCGTGCTTTGTTGT	CTTCGCACGTAGAGTTTGATGTTGT	1.04	EH038163.1
	HSP70	TTTGAGGAGCTGTGCATGGACTACTT	GGAACCACCCACCAGAACCACATCAT	1.03	EH037708.1
	Malonyl Co-A carboxylase	TTTCCAAAGGGCTTCTCGTGTGC	ACCCTTCTTCTCAGCCAGCTCCTTCAG	0.99	JF915367
	α-ketoglutarate dehydrogenase	ATCAAGATGCGAAAGGAATACAAG	GCATAAGCACTGGCCAGAAAGAAC	0.99	EH037114.1
	GAPDH	GCCCTGCGTGTGCCAACCAT	CTTCATCTCAGCGCAAATCTCCTCGTA		EH036392.1
	β-actin	TGGACAACGGAAGCGGAATG	GCCAACAATGGATGGGAAAACT	1.04	[24]
	PCNA	GAGTTTCAGAAGATTTGCCGAGAT	ACATTGCCACTGCCGAGGTC	0.98	[24]

### Statistical analysis

IPAM data was analysed using a generalised linear model using temperature and day as mains effects and temperature × day as an interaction. Changes in gene expression were determined using the program REST 2009 [60] (http://www.qiagen.com/products/rest2009software.aspx), with 10,000 iterations. For analysis of coral expression AdoHyclase and S7 were used as housekeeping genes after being found as the most stable housekeeping gene by GeNorm (http://medgen.ugent.be/~jvdesomp/genorm/) analysis of five identified by Seneca et al. [59] (GAPDH, rpL9, Ctg 1913). It was not possible to consistently amplify products using rpL13a or Ctg3235 primers. For *Symbiodinium* β-actin and proliferating cell nuclear antigen (PCNA) were used for analysis of gene expression [24].
